# Poly[octa­carbonyl­heptakis­(tetra­hydro­furan)­diironmanganesedisodium(2 *Mn*—*Fe*)]

**DOI:** 10.1107/S2414314621008452

**Published:** 2021-08-27

**Authors:** George N. Harakas, Bruce R. Whittlesey

**Affiliations:** a Department of Chemistry, PO Box 6949, Radford University, Radford, VA 24142, USA; b Department of Chemistry, 1204 Boston Avenue, Texas Tech University, Lubbock, TX 79409, USA; Sunway University, Malaysia

**Keywords:** crystal structure, metal cluster, iron, manganese

## Abstract

The reaction of Na_2_Fe(CO)_4_ with an excess of MnCl_2_ in tetra­hydro­furan (THF) produced {Fe_2_Mn(C_4_H_8_O)_2_Na_2_(C_4_H_8_O)_5_(CO)_8_]_*n*_ or C_36_H_56_Fe_2_MnNa_2_O_15_. The compound is a xenophilic dianion with short iron–manganese bond lengths of 2.6274 (10) and 2.6294 (10) Å. The sodium cations of the dianion are coordinated to THF ligands and have isocarbonyl inter­actions forming a polymeric (two-dimensional) structure in the crystal. One of the THF mol­ecules was modeled with the carbon atoms being statistically disordered.

## Abstract 2

The title compound is a xenophilic transition-metal cluster with short iron–manganese bonds of 2.6274 (10) and 2.6294 (10) Å. The complex forms a polymeric (two-dimensional) structure through isocarbonyl linkages between the sodium cations and the Fe(CO)_4_ fragments of the cluster.

## Graphical abstract

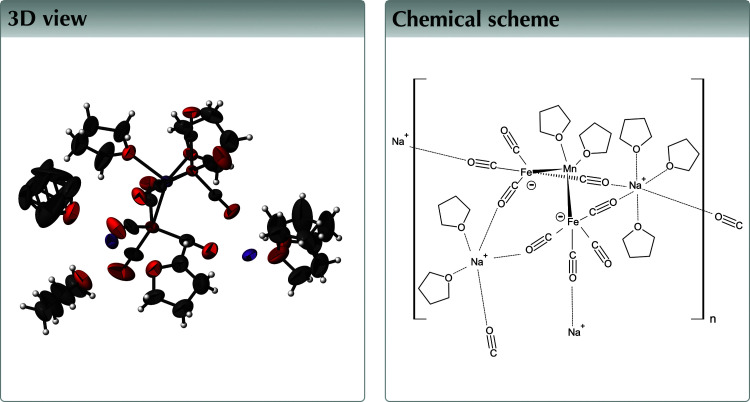

## Structure description

**Figure 1:**
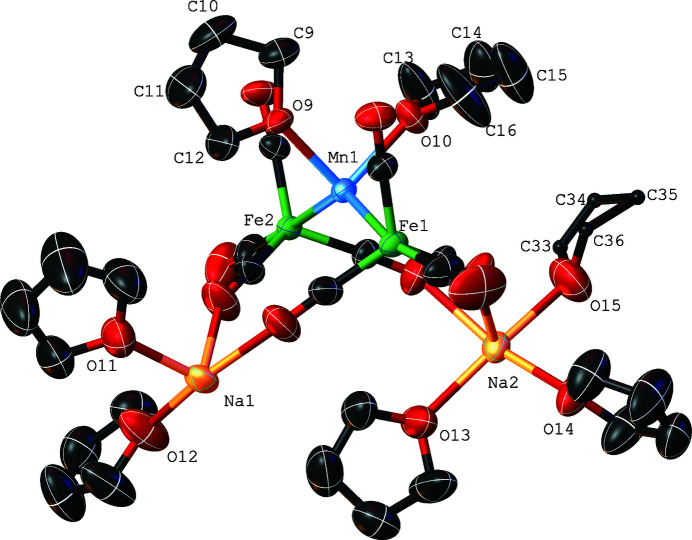
The asymmetric unit of the title compound, with displacement ellipsoids drawn at the 50% probability level. One tetra­hydro­furan mol­ecule coordinating Na2 is statistically disordered. For clarity, atoms C33–C36 are shown isotropically with a 50% probability level and atoms C33*A*–C36*A* are not shown.

**Figure 2:**
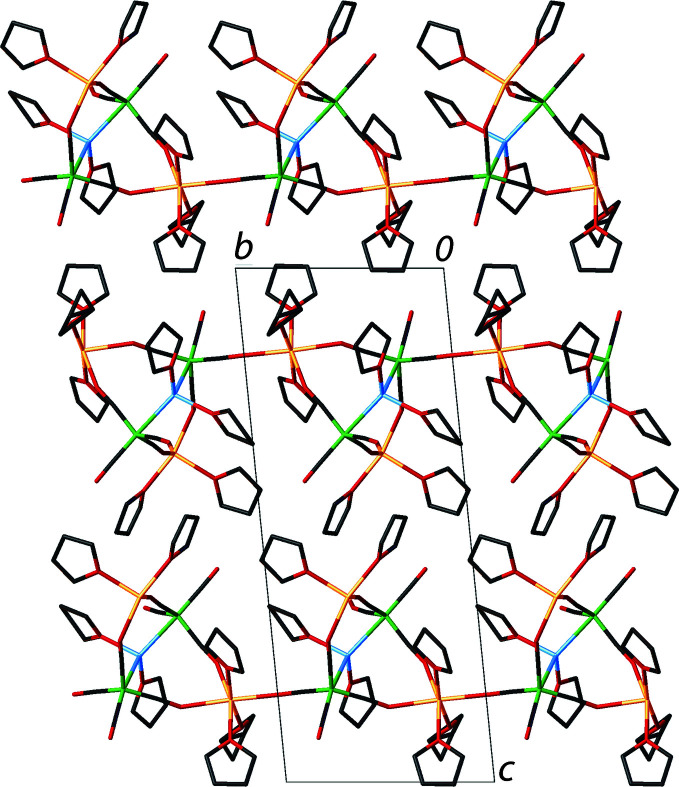
Packing diagram of the title compound, viewed along the *a* axis, highlighting the stacking of layers.

The term xenophilic transition-metal cluster was first used to describe the unusual structure of {μ-Mn(THF)_2_}Fe_2_(CO)_8_ (Harakas & Whittlesey, 1996 ▸). In a xenophilic cluster, two transition metals with open *d*-shells form a direct, unbridged bond between a metal center attached to π-acceptor ligands and one that is bound only to donor ligands that have no π-acceptor capabilities (Whittlesey, 2000 ▸). The title compound, Na_2_{μ-Mn(THF)_2_}Fe_2_(CO)_8_, has unsupported metal–metal bonds of 2.6274 (10) and 2.6294 (10) Å between the Mn(THF)_2_ and two Fe(CO)_4_ fragments (Fig. 1 ▸). The Mn—Fe bond lengths are significantly shorter than those of 2.813 (3) Å observed for FeMn_2_(CO)_14_ (Agron *et al.*,1967 ▸) and 2.841 (4) Å for (μ-C_5_H_5_)(OC)_2_FeMn(CO)_5_ (Hansen, *et al.*, 1966 ▸). The Fe—Mn bond distances for {μ-Mn(THF)_2_}Fe_2_(CO)_8_ are 2.633 (1) and 2.601 (1) Å (Harakas & Whittlesey, 1996 ▸). Despite the similarity of metal–metal bond lengths, the Fe—Mn—Fe bond angle of 136.81 (3)° for the dianion is considerable larger than 112.8 (1)°, which was observed for neutral {μ-Mn(THF)_2_}Fe_2_(CO)_8_. The sodium cations of the title complex are bound to the dianion *via* isocarbonyl linkages. The isocarbonyl linkages to the sodium cations create a two-dimensional network (Fig. 2 ▸).

## Synthesis and crystallization

All manipulations were conducted using inert atmosphere techniques. In a dry box Na_2_Fe(CO)_4_ (0.997 g, 4.66 mmol) and MnCl_2_ (1.181 g, 9.384 mmol) were added to a 150 mL Schlenk flask with a magnetic stirring bar. The flask was closed with a rubber septum then transferred to a Schlenk line. To this flask, 75 mL of anhydrous THF were added and the reaction mixture was stirred rapidly. The solution formed a deep yellow–orange color as the THF was added. The mixture was stirred for 30 minutes then allowed to settle for 24 h. The bright-orange solution was deca­nted from the solids into another Schlenk flask. The solution was placed into a freezer at −15°C. Large block-like yellow crystals formed after several days. At 25 °C the complex rapidly decomposes when exposed to oxygen and when the crystals are removed from the mother liquor. A single crystal was coated with NVH oil and mounted on a MiTeGen loop under a stream of argon gas then cooled to −45°C for data collection.

## Refinement

Crystal data, data collection, and structure refinement details are summarized in Table 1 ▸. One tetra­hydro­furan mol­ecule coordinating Na2 was modeled for disorder with the C33—C36 atoms statistically disordered. The H atoms on the disordered THF molecule C33–C36/C33*A*–C36*A* were not located.

## Supplementary Material

Crystal structure: contains datablock(s) global, I. DOI: 10.1107/S2414314621008452/tk4070sup1.cif

Structure factors: contains datablock(s) I. DOI: 10.1107/S2414314621008452/tk4070Isup2.hkl

CCDC reference: 2103160

Additional supporting information:  crystallographic information; 3D view; checkCIF report

## Figures and Tables

**Table 1:** Experimental details

Crystal data	
Chemical formula	[Fe_2_MnNa_2_(C_4_H_8_O)_7_(CO)_8_]
*M*_r_	933.36
Crystal system, space group	Triclinic, *P* 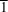
Temperature (K)	228
*a*, *b*, *c* (Å)	9.3839 (11), 9.9442 (11), 24.348 (3)
α, β, γ (°)	95.154 (5), 92.640 (5), 99.414 (5)
*V* (Å^3^)	2228.1 (4)
*Z*	2
Radiation type	Mo *K*α
μ (mm^−1^)	1.00
Crystal size (mm)	0.39 × 0.35 × 0.31

Data collection	
Diffractometer	Bruker D8 Quest Eco, Photon PII 7
Absorption correction	Multi-scan (*SADABS*; Krause *et al.*, 2015 ▸)
*T*_min_, *T*_max_	0.68, 0.73
No. of measured, independent and observed [*I* > 2σ(*I*)] reflections	154320, 12761, 10027
*R*_int_	0.057
(sin θ/λ)_max_ (Å^−1^)	0.701

Refinement	
*R*[*F* ^2^ > 2σ(*F* ^2^)], *wR*(*F* ^2^), *S*	0.090, 0.217, 1.12
No. of reflections	12761
No. of parameters	541
H-atom treatment	H-atom parameters constrained
Δρ_max_, Δρ_min_ (e Å^−3^)	0.89, −1.78

Computer programs: *APEX3* and *SAINT* (Bruker, 2019 ▸), *SHELXT2018/2* (Sheldrick, 2015*a* ▸), *SHELXL2018/2* (Sheldrick, 2015*b* ▸), *OLEX2* (Dolomanov *et al.*, 2009 ▸), and *shelXle* (Hübschle *et al.*, 2011 ▸).

## full crystallographic data

### Crystal data

**Table:**

[Fe_2_MnNa_2_(C_4_H_8_O)_7_(CO)_8_]	*Z* = 2
*M**_r_* = 933.36	*F*(000) = 966
Triclinic, *P*1	*D*_x_ = 1.391 Mg m^−^^3^
*a* = 9.3839 (11) Å	Mo *K*α radiation, λ = 0.71073 Å
*b* = 9.9442 (11) Å	Cell parameters from 9427 reflections
*c* = 24.348 (3) Å	θ = 2.8–29.7°
α = 95.154 (5)°	µ = 1.00 mm^−^^1^
β = 92.640 (5)°	*T* = 228 K
γ = 99.414 (5)°	Cube, yellow
*V* = 2228.1 (4) Å^3^	0.39 × 0.35 × 0.31 mm

### Data collection

**Table:**

Bruker D8 Quest Eco, Photon PII 7 diffractometer	10027 reflections with *I* > 2σ(*I*)
Detector resolution: 7.3910 pixels mm^-1^	*R*_int_ = 0.057
phi and ω scans	θ_max_ = 29.9°, θ_min_ = 2.3°
Absorption correction: multi-scan (SADABS; Krause *et al.*, 2015)	*h* = −13→13
*T*_min_ = 0.68, *T*_max_ = 0.73	*k* = −13→13
154320 measured reflections	*l* = −34→34
12761 independent reflections	

### Refinement

**Table:**

Refinement on *F*^2^	0 restraints
Least-squares matrix: full	Hydrogen site location: inferred from neighbouring sites
*R*[*F*^2^ > 2σ(*F*^2^)] = 0.090	H-atom parameters constrained
*wR*(*F*^2^) = 0.217	*w* = 1/[σ^2^(*F*_o_^2^) + (0.0431*P*)^2^ + 11.8029*P*] where *P* = (*F*_o_^2^ + 2*F*_c_^2^)/3
*S* = 1.12	(Δ/σ)_max_ = 0.001
12761 reflections	Δρ_max_ = 0.89 e Å^−^^3^
541 parameters	Δρ_min_ = −1.77 e Å^−^^3^

### Special details

**Table:**

Geometry. All esds (except the esd in the dihedral angle between two l.s. planes) are estimated using the full covariance matrix. The cell esds are taken into account individually in the estimation of esds in distances, angles and torsion angles; correlations between esds in cell parameters are only used when they are defined by crystal symmetry. An approximate (isotropic) treatment of cell esds is used for estimating esds involving l.s. planes.

### Fractional atomic coordinates and isotropic or equivalent isotropic displacement parameters (Å^2^)

**Table:**

	*x*	*y*	*z*	*U*_iso_*/*U*_eq_	Occ. (<1)
Fe1	0.48239 (8)	0.26076 (7)	0.18023 (3)	0.03809 (18)	
Fe2	0.79486 (7)	0.55567 (7)	0.32428 (3)	0.03322 (16)	
Mn1	0.71446 (8)	0.35449 (7)	0.24588 (3)	0.03290 (16)	
Na1	0.2691 (2)	0.3843 (3)	0.36059 (10)	0.0526 (6)	
Na2	0.6115 (3)	0.7720 (2)	0.16011 (9)	0.0469 (5)	
O1	0.7818 (6)	0.7222 (5)	0.23192 (19)	0.0660 (12)	
O2	0.8840 (8)	0.7683 (5)	0.4142 (2)	0.0891 (19)	
O3	0.5154 (5)	0.4355 (7)	0.3595 (2)	0.0789 (16)	
O4	1.0325 (4)	0.4044 (5)	0.33895 (19)	0.0599 (11)	
O5	0.5983 (7)	0.5281 (5)	0.1459 (2)	0.0813 (17)	
O6	0.6271 (5)	0.0226 (4)	0.17057 (19)	0.0570 (11)	
O7	0.3016 (6)	0.2677 (6)	0.2747 (2)	0.0799 (16)	
O8	0.2773 (8)	0.1757 (8)	0.0860 (3)	0.121 (3)	
O9	0.7649 (4)	0.1760 (4)	0.28376 (16)	0.0454 (8)	
O10	0.8976 (5)	0.3564 (5)	0.19223 (17)	0.0568 (10)	
O11	0.2429 (7)	0.1979 (6)	0.4098 (2)	0.0818 (16)	
O12	0.2849 (6)	0.5508 (7)	0.4348 (3)	0.095 (2)	
O13	0.4276 (6)	0.7701 (6)	0.2220 (2)	0.0802 (16)	
O14	0.4509 (7)	0.7502 (5)	0.0810 (2)	0.0772 (15)	
O15	0.8067 (8)	0.8112 (8)	0.1062 (3)	0.103 (2)	
C1	0.7869 (6)	0.6505 (6)	0.2674 (2)	0.0420 (11)	
C2	0.8491 (8)	0.6852 (6)	0.3783 (2)	0.0539 (15)	
C3	0.6276 (6)	0.4794 (7)	0.3437 (2)	0.0492 (13)	
C4	0.9358 (5)	0.4632 (5)	0.3307 (2)	0.0383 (10)	
C5	0.5563 (7)	0.4215 (6)	0.1616 (2)	0.0520 (14)	
C6	0.5742 (6)	0.1216 (5)	0.1755 (2)	0.0399 (10)	
C7	0.3776 (7)	0.2664 (7)	0.2378 (2)	0.0527 (14)	
C8	0.3572 (7)	0.2088 (7)	0.1237 (3)	0.0628 (17)	
C9	0.8819 (7)	0.0999 (6)	0.2771 (3)	0.0598 (16)	
H9A	0.973803	0.161725	0.275033	0.072*	
H9B	0.864289	0.036212	0.243395	0.072*	
C10	0.8842 (10)	0.0245 (8)	0.3269 (4)	0.086 (3)	
H10A	0.94729	0.078928	0.356835	0.103*	
H10B	0.918117	−0.062826	0.318562	0.103*	
C11	0.7309 (13)	0.0016 (10)	0.3420 (4)	0.106 (3)	
H11A	0.678883	−0.085972	0.324062	0.127*	
H11B	0.725456	0.001604	0.382053	0.127*	
C12	0.6697 (9)	0.1166 (9)	0.3220 (4)	0.079 (2)	
H12A	0.573156	0.08388	0.304011	0.095*	
H12B	0.661356	0.184459	0.352987	0.095*	
C13	1.0337 (9)	0.4396 (13)	0.2019 (3)	0.111 (4)	
H13A	1.095579	0.396952	0.226162	0.133*	
H13B	1.023152	0.528918	0.220231	0.133*	
C14	1.0999 (10)	0.4576 (12)	0.1496 (4)	0.096 (3)	
H14A	1.110652	0.553584	0.141814	0.115*	
H14B	1.195631	0.430306	0.150653	0.115*	
C15	1.0039 (12)	0.3712 (15)	0.1082 (4)	0.129 (5)	
H15A	1.056239	0.308318	0.087191	0.155*	
H15B	0.962303	0.426854	0.082469	0.155*	
C16	0.8913 (12)	0.2957 (12)	0.1373 (4)	0.129 (5)	
H16A	0.796391	0.297377	0.119036	0.155*	
H16B	0.904888	0.199993	0.137059	0.155*	
C17	0.1889 (12)	0.1797 (12)	0.4620 (4)	0.103 (3)	
H17A	0.084358	0.179603	0.460333	0.124*	
H17B	0.235543	0.25449	0.489131	0.124*	
C18	0.2193 (18)	0.0494 (12)	0.4780 (5)	0.140 (5)	
H18A	0.290831	0.063918	0.509484	0.168*	
H18B	0.130854	−0.007246	0.488238	0.168*	
C19	0.274 (2)	−0.0141 (13)	0.4315 (6)	0.178 (8)	
H19A	0.368185	−0.038239	0.441643	0.214*	
H19B	0.207838	−0.098531	0.41783	0.214*	
C20	0.2889 (13)	0.0753 (10)	0.3900 (4)	0.104 (3)	
H20A	0.22986	0.033685	0.356656	0.125*	
H20B	0.390167	0.094416	0.380624	0.125*	
C21	0.1654 (10)	0.5705 (13)	0.4651 (4)	0.114 (4)	
H21A	0.108313	0.481587	0.470617	0.137*	
H21B	0.103597	0.621606	0.444536	0.137*	
C22	0.2130 (12)	0.6441 (13)	0.5170 (4)	0.114 (4)	
H22A	0.19841	0.583595	0.546576	0.137*	
H22B	0.158879	0.719473	0.524342	0.137*	
C23	0.3688 (11)	0.6981 (10)	0.5141 (4)	0.090 (3)	
H23A	0.42569	0.671067	0.544781	0.108*	
H23B	0.385726	0.798283	0.515469	0.108*	
C24	0.4078 (10)	0.6345 (10)	0.4593 (4)	0.088 (3)	
H24A	0.442022	0.706119	0.435602	0.105*	
H24B	0.485049	0.580785	0.465058	0.105*	
C25	0.3947 (9)	0.6781 (9)	0.2624 (3)	0.076 (2)	
H25A	0.482244	0.670929	0.284741	0.091*	
H25B	0.352261	0.586772	0.244962	0.091*	
C26	0.2899 (12)	0.7358 (13)	0.2970 (4)	0.108 (3)	
H26A	0.339321	0.793617	0.329264	0.129*	
H26B	0.219405	0.662848	0.309665	0.129*	
C27	0.2170 (10)	0.8196 (11)	0.2590 (4)	0.094 (3)	
H27A	0.121279	0.770332	0.245063	0.113*	
H27B	0.205903	0.90761	0.278391	0.113*	
C28	0.3118 (9)	0.8401 (11)	0.2142 (5)	0.104 (4)	
H28A	0.258339	0.805669	0.178838	0.125*	
H28B	0.347993	0.938086	0.213421	0.125*	
C29	0.3942 (12)	0.6280 (9)	0.0499 (4)	0.097 (3)	
H29A	0.313456	0.579395	0.068406	0.116*	
H29B	0.468602	0.569649	0.046549	0.116*	
C30	0.3450 (16)	0.6546 (11)	−0.0033 (4)	0.129 (5)	
H30A	0.243118	0.613051	−0.010931	0.155*	
H30B	0.401758	0.615319	−0.031526	0.155*	
C31	0.3622 (11)	0.8036 (10)	−0.0046 (3)	0.089 (3)	
H31A	0.267887	0.833208	−0.00809	0.107*	
H31B	0.419165	0.833161	−0.035416	0.107*	
C32	0.4391 (13)	0.8591 (9)	0.0489 (4)	0.108 (4)	
H32A	0.535733	0.907845	0.042802	0.13*	
H32B	0.385772	0.923916	0.068191	0.13*	
C33	0.947 (3)	0.872 (4)	0.1287 (10)	0.118 (10)	0.5
C34	1.020 (5)	0.819 (9)	0.094 (4)	0.31 (4)	0.5
C33A	0.876 (5)	0.924 (3)	0.085 (2)	0.184 (19)	0.5
C35	0.963 (3)	0.752 (3)	0.0441 (12)	0.111 (8)	0.5
C34A	1.037 (4)	0.884 (5)	0.0834 (15)	0.20 (3)	0.5
C36	0.849 (6)	0.705 (3)	0.080 (3)	0.27 (3)	0.5
C35A	0.952 (3)	0.862 (4)	0.0364 (10)	0.111 (8)	0.5
C36A	0.826 (3)	0.782 (5)	0.0497 (8)	0.167 (18)	0.5

### Atomic displacement parameters (Å^2^)

**Table:**

	*U*^11^	*U*^22^	*U*^33^	*U*^12^	*U*^13^	*U*^23^
Fe1	0.0406 (4)	0.0376 (4)	0.0362 (3)	0.0126 (3)	−0.0065 (3)	−0.0024 (3)
Fe2	0.0342 (3)	0.0371 (3)	0.0296 (3)	0.0117 (3)	0.0000 (2)	0.0004 (2)
Mn1	0.0361 (4)	0.0339 (3)	0.0300 (3)	0.0113 (3)	−0.0008 (3)	0.0019 (3)
Na1	0.0336 (10)	0.0715 (15)	0.0541 (13)	0.0139 (10)	0.0075 (9)	0.0017 (11)
Na2	0.0604 (13)	0.0418 (11)	0.0404 (11)	0.0134 (10)	0.0029 (9)	0.0065 (9)
O1	0.079 (3)	0.063 (3)	0.055 (3)	0.005 (2)	−0.009 (2)	0.022 (2)
O2	0.158 (6)	0.055 (3)	0.052 (3)	0.028 (3)	−0.017 (3)	−0.016 (2)
O3	0.037 (2)	0.131 (5)	0.065 (3)	0.005 (3)	0.016 (2)	0.002 (3)
O4	0.043 (2)	0.074 (3)	0.066 (3)	0.028 (2)	−0.0087 (19)	−0.008 (2)
O5	0.130 (5)	0.041 (2)	0.068 (3)	0.004 (3)	−0.031 (3)	0.016 (2)
O6	0.059 (3)	0.038 (2)	0.073 (3)	0.0169 (18)	−0.007 (2)	−0.0112 (19)
O7	0.065 (3)	0.119 (5)	0.059 (3)	0.028 (3)	0.017 (2)	−0.003 (3)
O8	0.113 (5)	0.135 (6)	0.103 (5)	0.043 (4)	−0.067 (4)	−0.048 (4)
O9	0.051 (2)	0.0416 (19)	0.048 (2)	0.0212 (16)	−0.0018 (16)	0.0069 (16)
O10	0.049 (2)	0.072 (3)	0.048 (2)	0.009 (2)	0.0145 (18)	−0.001 (2)
O11	0.100 (4)	0.085 (4)	0.069 (3)	0.028 (3)	0.029 (3)	0.022 (3)
O12	0.055 (3)	0.127 (5)	0.089 (4)	0.005 (3)	0.007 (3)	−0.042 (4)
O13	0.078 (3)	0.092 (4)	0.088 (4)	0.040 (3)	0.033 (3)	0.041 (3)
O14	0.120 (5)	0.057 (3)	0.053 (3)	0.022 (3)	−0.025 (3)	0.001 (2)
O15	0.110 (5)	0.114 (5)	0.094 (4)	0.027 (4)	0.057 (4)	0.022 (4)
C1	0.044 (3)	0.046 (3)	0.036 (2)	0.009 (2)	−0.005 (2)	0.002 (2)
C2	0.082 (4)	0.042 (3)	0.039 (3)	0.021 (3)	−0.009 (3)	−0.002 (2)
C3	0.036 (3)	0.071 (4)	0.042 (3)	0.016 (3)	0.002 (2)	0.004 (3)
C4	0.030 (2)	0.042 (3)	0.041 (2)	0.0052 (19)	0.0004 (18)	−0.005 (2)
C5	0.076 (4)	0.043 (3)	0.037 (3)	0.020 (3)	−0.016 (3)	−0.001 (2)
C6	0.046 (3)	0.031 (2)	0.041 (2)	0.008 (2)	−0.004 (2)	−0.0037 (19)
C7	0.049 (3)	0.062 (4)	0.049 (3)	0.020 (3)	−0.003 (3)	−0.004 (3)
C8	0.059 (4)	0.063 (4)	0.062 (4)	0.019 (3)	−0.026 (3)	−0.016 (3)
C9	0.059 (4)	0.042 (3)	0.079 (4)	0.017 (3)	−0.008 (3)	0.004 (3)
C10	0.089 (6)	0.064 (5)	0.110 (7)	0.029 (4)	−0.027 (5)	0.020 (4)
C11	0.139 (9)	0.091 (7)	0.106 (7)	0.050 (6)	0.031 (7)	0.050 (6)
C12	0.088 (5)	0.086 (5)	0.082 (5)	0.039 (4)	0.033 (4)	0.045 (4)
C13	0.060 (5)	0.193 (11)	0.061 (5)	−0.025 (6)	0.017 (4)	−0.015 (6)
C14	0.074 (5)	0.139 (9)	0.071 (5)	−0.008 (5)	0.021 (4)	0.022 (5)
C15	0.091 (7)	0.220 (14)	0.060 (5)	−0.011 (8)	0.015 (5)	−0.018 (7)
C16	0.118 (8)	0.143 (9)	0.094 (7)	−0.043 (7)	0.054 (6)	−0.069 (7)
C17	0.122 (8)	0.121 (8)	0.083 (6)	0.047 (7)	0.044 (6)	0.029 (6)
C18	0.237 (16)	0.101 (8)	0.103 (8)	0.056 (9)	0.075 (10)	0.040 (7)
C19	0.34 (2)	0.095 (9)	0.129 (11)	0.083 (12)	0.087 (14)	0.034 (8)
C20	0.137 (9)	0.084 (6)	0.097 (7)	0.031 (6)	0.038 (6)	0.005 (5)
C21	0.063 (5)	0.160 (10)	0.105 (7)	0.013 (6)	0.010 (5)	−0.055 (7)
C22	0.105 (8)	0.150 (10)	0.068 (5)	−0.015 (7)	0.020 (5)	−0.029 (6)
C23	0.103 (7)	0.088 (6)	0.070 (5)	0.006 (5)	−0.018 (5)	−0.004 (4)
C24	0.075 (5)	0.089 (6)	0.094 (6)	0.014 (5)	−0.005 (5)	−0.013 (5)
C25	0.070 (5)	0.080 (5)	0.083 (5)	0.013 (4)	0.016 (4)	0.033 (4)
C26	0.097 (7)	0.154 (10)	0.074 (6)	0.013 (7)	0.014 (5)	0.028 (6)
C27	0.073 (5)	0.105 (7)	0.105 (7)	0.024 (5)	0.019 (5)	−0.005 (6)
C28	0.063 (5)	0.123 (8)	0.148 (9)	0.045 (5)	0.032 (5)	0.065 (7)
C29	0.134 (8)	0.062 (5)	0.083 (6)	−0.002 (5)	−0.035 (6)	0.003 (4)
C30	0.213 (14)	0.100 (8)	0.071 (6)	0.042 (8)	−0.055 (7)	−0.016 (5)
C31	0.106 (7)	0.100 (7)	0.061 (5)	0.019 (5)	−0.008 (4)	0.018 (4)
C32	0.159 (10)	0.060 (5)	0.099 (7)	0.020 (5)	−0.056 (7)	0.003 (5)
C33	0.078 (14)	0.18 (3)	0.082 (14)	−0.008 (16)	0.030 (11)	−0.031 (15)
C34	0.14 (4)	0.46 (11)	0.38 (10)	0.15 (6)	−0.09 (5)	0.12 (9)
C33A	0.22 (4)	0.074 (15)	0.28 (5)	0.04 (2)	0.17 (4)	0.03 (2)
C35	0.084 (15)	0.14 (2)	0.104 (17)	0.014 (15)	0.041 (13)	−0.031 (16)
C34A	0.14 (3)	0.28 (5)	0.12 (2)	−0.12 (3)	0.08 (2)	−0.08 (3)
C36	0.30 (5)	0.077 (16)	0.47 (7)	0.04 (2)	0.32 (6)	0.02 (3)
C35A	0.13 (2)	0.14 (2)	0.070 (13)	0.006 (18)	0.040 (13)	0.036 (15)
C36A	0.092 (17)	0.34 (5)	0.049 (10)	−0.03 (3)	−0.002 (10)	0.017 (19)

### Geometric parameters (Å, º)

**Table:**

Fe1—C6	1.743 (5)	O15—C36	1.31 (3)
Fe1—C5	1.744 (6)	O15—C33A	1.36 (3)
Fe1—C8	1.750 (6)	O15—C36A	1.41 (2)
Fe1—C7	1.752 (6)	O15—C33	1.42 (3)
Fe1—Mn1	2.6294 (10)	C9—C10	1.483 (10)
Fe2—C3	1.733 (6)	C10—C11	1.488 (13)
Fe2—C4	1.739 (5)	C11—C12	1.470 (10)
Fe2—C1	1.749 (5)	C13—C14	1.454 (11)
Fe2—C2	1.750 (6)	C14—C15	1.440 (13)
Fe2—Mn1	2.6274 (10)	C15—C16	1.445 (13)
Mn1—O9	2.180 (4)	C17—C18	1.454 (14)
Mn1—O10	2.205 (4)	C18—C19	1.405 (15)
Na1—O11	2.283 (6)	C19—C20	1.402 (14)
Na1—O3	2.285 (5)	C21—C22	1.416 (12)
Na1—O4^i^	2.303 (4)	C22—C23	1.479 (13)
Na1—O12	2.321 (6)	C23—C24	1.512 (12)
Na1—O7	2.355 (5)	C25—C26	1.477 (13)
Na2—O15	2.305 (6)	C26—C27	1.512 (13)
Na2—O13	2.341 (5)	C27—C28	1.446 (12)
Na2—O14	2.362 (5)	C29—C30	1.418 (12)
Na2—O5	2.401 (5)	C30—C31	1.468 (13)
Na2—O1	2.452 (5)	C31—C32	1.474 (11)
Na2—O6^ii^	2.462 (4)	C33—C34	1.25 (8)
O1—C1	1.173 (6)	C33—C33A	1.41 (5)
O2—C2	1.145 (7)	C33—C34A	1.42 (3)
O3—C3	1.168 (7)	C33—C36	2.01 (6)
O4—C4	1.176 (6)	C34—C34A	0.71 (9)
O5—C5	1.172 (7)	C34—C35	1.36 (8)
O6—C6	1.173 (6)	C34—C35A	1.62 (6)
O7—C7	1.173 (7)	C34—C36	1.80 (8)
O8—C8	1.147 (7)	C34—C33A	1.85 (6)
O9—C12	1.421 (8)	C34—C36A	2.03 (5)
O9—C9	1.438 (7)	C33A—C35A	1.54 (4)
O10—C13	1.400 (9)	C33A—C36A	1.58 (5)
O10—C16	1.412 (9)	C33A—C34A	1.62 (6)
O11—C17	1.405 (9)	C35—C35A	1.14 (3)
O11—C20	1.411 (10)	C35—C36A	1.37 (4)
O12—C24	1.383 (10)	C35—C36	1.46 (4)
O12—C21	1.400 (10)	C35—C34A	1.59 (4)
O13—C28	1.398 (9)	C34A—C35A	1.34 (5)
O13—C25	1.415 (8)	C36—C36A	1.15 (6)
O14—C29	1.386 (9)	C35A—C36A	1.38 (4)
O14—C32	1.407 (9)		
			
C6—Fe1—C5	122.8 (3)	O13—C25—C26	105.7 (7)
C6—Fe1—C8	98.7 (3)	C25—C26—C27	103.9 (7)
C5—Fe1—C8	98.5 (3)	C28—C27—C26	105.3 (7)
C6—Fe1—C7	113.2 (3)	O13—C28—C27	109.0 (8)
C5—Fe1—C7	114.0 (3)	O14—C29—C30	109.8 (8)
C8—Fe1—C7	104.9 (3)	C29—C30—C31	108.0 (8)
C6—Fe1—Mn1	78.41 (16)	C30—C31—C32	104.0 (7)
C5—Fe1—Mn1	72.21 (18)	O14—C32—C31	109.0 (7)
C8—Fe1—Mn1	165.8 (3)	C34—C33—C33A	88 (3)
C7—Fe1—Mn1	88.9 (2)	C34—C33—O15	99 (3)
C3—Fe2—C4	116.5 (3)	C33A—C33—O15	57.5 (18)
C3—Fe2—C1	114.4 (3)	C34—C33—C34A	30 (4)
C4—Fe2—C1	118.3 (2)	C33A—C33—C34A	70 (3)
C3—Fe2—C2	102.2 (3)	O15—C33—C34A	106 (2)
C4—Fe2—C2	99.4 (3)	C34—C33—C36	62 (4)
C1—Fe2—C2	101.7 (3)	C33A—C33—C36	75 (2)
C3—Fe2—Mn1	77.0 (2)	O15—C33—C36	40.4 (15)
C4—Fe2—Mn1	79.23 (16)	C34A—C33—C36	81.2 (19)
C1—Fe2—Mn1	80.45 (17)	C34A—C34—C33	88 (9)
C2—Fe2—Mn1	177.86 (19)	C34A—C34—C35	95 (8)
O9—Mn1—O10	92.17 (16)	C33—C34—C35	124 (3)
O9—Mn1—Fe2	103.15 (11)	C34A—C34—C35A	54 (6)
O10—Mn1—Fe2	106.01 (12)	C33—C34—C35A	102 (3)
O9—Mn1—Fe1	104.77 (11)	C35—C34—C35A	44 (2)
O10—Mn1—Fe1	105.15 (12)	C34A—C34—C36	125 (6)
Fe2—Mn1—Fe1	136.81 (3)	C33—C34—C36	80 (4)
O11—Na1—O3	101.2 (2)	C35—C34—C36	53 (3)
O11—Na1—O4^i^	102.2 (2)	C35A—C34—C36	75 (3)
O3—Na1—O4^i^	156.5 (2)	C34A—C34—C33A	60 (5)
O11—Na1—O12	97.5 (3)	C33—C34—C33A	50 (2)
O3—Na1—O12	87.6 (2)	C35—C34—C33A	84 (3)
O4^i^—Na1—O12	91.3 (2)	C35A—C34—C33A	52 (2)
O11—Na1—O7	96.9 (2)	C36—C34—C33A	72 (2)
O3—Na1—O7	80.4 (2)	C34A—C34—C36A	91 (6)
O4^i^—Na1—O7	94.89 (19)	C33—C34—C36A	82 (2)
O12—Na1—O7	162.8 (3)	C35—C34—C36A	42.3 (17)
O15—Na2—O13	170.2 (3)	C35A—C34—C36A	42.7 (17)
O15—Na2—O14	90.4 (3)	C36—C34—C36A	34 (2)
O13—Na2—O14	94.0 (2)	C33A—C34—C36A	47.7 (19)
O15—Na2—O5	92.4 (3)	O15—C33A—C33	61.4 (19)
O13—Na2—O5	96.5 (2)	O15—C33A—C35A	103 (2)
O14—Na2—O5	86.82 (18)	C33—C33A—C35A	99 (2)
O15—Na2—O1	86.8 (2)	O15—C33A—C36A	56.7 (15)
O13—Na2—O1	91.5 (2)	C33—C33A—C36A	96 (2)
O14—Na2—O1	162.5 (2)	C35A—C33A—C36A	52.6 (19)
O5—Na2—O1	76.04 (17)	O15—C33A—C34A	99 (3)
O15—Na2—O6^ii^	85.7 (2)	C33—C33A—C34A	55 (3)
O13—Na2—O6^ii^	85.38 (19)	C35A—C33A—C34A	50.1 (17)
O14—Na2—O6^ii^	91.60 (17)	C36A—C33A—C34A	85 (2)
O5—Na2—O6^ii^	177.6 (2)	O15—C33A—C34	77 (3)
O1—Na2—O6^ii^	105.41 (17)	C33—C33A—C34	43 (3)
C1—O1—Na2	140.3 (4)	C35A—C33A—C34	56 (3)
C3—O3—Na1	158.0 (5)	C36A—C33A—C34	72 (3)
C4—O4—Na1^iii^	155.7 (4)	C34A—C33A—C34	22 (3)
C5—O5—Na2	146.8 (5)	C35A—C35—C34	80 (4)
C6—O6—Na2^iv^	151.9 (4)	C35A—C35—C36A	66 (3)
C7—O7—Na1	143.9 (5)	C34—C35—C36A	96 (3)
C12—O9—C9	109.7 (5)	C35A—C35—C36	107 (2)
C12—O9—Mn1	118.7 (4)	C34—C35—C36	79 (4)
C9—O9—Mn1	131.6 (4)	C36A—C35—C36	48 (2)
C13—O10—C16	106.6 (6)	C35A—C35—C34A	56 (3)
C13—O10—Mn1	126.2 (4)	C34—C35—C34A	26 (4)
C16—O10—Mn1	126.2 (5)	C36A—C35—C34A	93 (3)
C17—O11—C20	107.7 (7)	C36—C35—C34A	97 (3)
C17—O11—Na1	130.8 (6)	C34—C34A—C35A	100 (7)
C20—O11—Na1	121.4 (5)	C34—C34A—C33	62 (7)
C24—O12—C21	109.6 (7)	C35A—C34A—C33	109 (3)
C24—O12—Na1	127.9 (5)	C34—C34A—C35	59 (8)
C21—O12—Na1	122.3 (5)	C35A—C34A—C35	45.0 (17)
C28—O13—C25	109.1 (6)	C33—C34A—C35	100 (2)
C28—O13—Na2	122.1 (5)	C34—C34A—C33A	98 (6)
C25—O13—Na2	127.1 (4)	C35A—C34A—C33A	62 (3)
C29—O14—C32	108.7 (6)	C33—C34A—C33A	55 (2)
C29—O14—Na2	125.1 (5)	C35—C34A—C33A	86 (3)
C32—O14—Na2	123.0 (5)	C36A—C36—O15	70 (2)
C36—O15—C33A	106.7 (18)	C36A—C36—C35	62 (3)
C36—O15—C36A	50 (3)	O15—C36—C35	109 (2)
C33A—O15—C36A	69 (2)	C36A—C36—C34	84 (3)
C36—O15—C33	95 (3)	O15—C36—C34	80 (4)
C33A—O15—C33	61 (2)	C35—C36—C34	48 (3)
C36A—O15—C33	103.9 (14)	C36A—C36—C33	85 (3)
C36—O15—Na2	118.1 (13)	O15—C36—C33	44.7 (19)
C33A—O15—Na2	133.7 (12)	C35—C36—C33	82 (2)
C36A—O15—Na2	133.8 (12)	C34—C36—C33	38 (3)
C33—O15—Na2	122.2 (10)	C35—C35A—C34A	79 (3)
O1—C1—Fe2	175.2 (5)	C35—C35A—C36A	65 (3)
O2—C2—Fe2	178.9 (6)	C34A—C35A—C36A	104.7 (19)
O3—C3—Fe2	175.4 (6)	C35—C35A—C33A	108 (2)
O4—C4—Fe2	175.3 (5)	C34A—C35A—C33A	68 (3)
O5—C5—Fe1	175.1 (5)	C36A—C35A—C33A	65 (2)
O6—C6—Fe1	175.2 (5)	C35—C35A—C34	56 (3)
O7—C7—Fe1	176.7 (6)	C34A—C35A—C34	25 (4)
O8—C8—Fe1	178.7 (9)	C36A—C35A—C34	84 (3)
O9—C9—C10	104.7 (6)	C33A—C35A—C34	72 (3)
C9—C10—C11	104.1 (6)	C36—C36A—C35	70 (3)
C12—C11—C10	105.0 (7)	C36—C36A—C35A	112 (4)
O9—C12—C11	107.2 (7)	C35—C36A—C35A	49.1 (16)
O10—C13—C14	109.5 (7)	C36—C36A—O15	60.5 (19)
C15—C14—C13	106.0 (8)	C35—C36A—O15	109 (2)
C14—C15—C16	106.5 (8)	C35A—C36A—O15	109 (3)
O10—C16—C15	108.8 (7)	C36—C36A—C33A	102 (4)
O11—C17—C18	108.0 (8)	C35—C36A—C33A	96 (2)
C19—C18—C17	105.9 (9)	C35A—C36A—C33A	62 (2)
C20—C19—C18	109.5 (10)	O15—C36A—C33A	53.9 (17)
C19—C20—O11	108.3 (9)	C36—C36A—C34	62 (3)
O12—C21—C22	109.8 (8)	C35—C36A—C34	42 (3)
C21—C22—C23	106.8 (8)	C35A—C36A—C34	53 (3)
C22—C23—C24	104.5 (7)	O15—C36A—C34	70 (3)
O12—C24—C23	107.7 (7)	C33A—C36A—C34	60 (3)

Symmetry codes: (i) *x*−1, *y*, *z*; (ii) *x*, *y*+1, *z*; (iii) *x*+1, *y*, *z*; (iv) *x*, *y*−1, *z*.
